# Outbreak preparedness for women and girls in low- and middle-income countries: a qualitative study

**DOI:** 10.1186/s44263-026-00305-7

**Published:** 2026-07-28

**Authors:** Danielle Sarraf, Melissa Meinhart, Ilana Seff, Catherine Poulton, Sarah Karmin, Christine Heckman, Lindsay Stark

**Affiliations:** 1 Bursky School of Public Health, Washington University in St. Louis, 1 Brookings Dr, St. Louis, MO 63130 USA; 2 The United Nations International Children’s Fund (UNICEF), 3 United Nations Plaza, New York, NY 10017 USA

**Keywords:** Infectious disease outbreaks, Low- and middle-income countries, Gender-based violence, Women and girls, Programming

## Abstract

**Background:**

Infectious disease outbreaks (“outbreaks”) pose heightened gender-based violence (GBV) risk for women and girls. However, little is known about the integration of GBV and gender-informed programming within outbreak preparedness and response in low- and middle-income countries (LMICs).

**Methods:**

This qualitative study draws on in-depth interviews with ten public health practitioners with experience supporting infectious disease outbreak preparedness and response in LMICs. Participants represented intergovernmental organizations and worked across multiple outbreak contexts, including Ebola, COVID-19, Mpox, Zika, and Cholera, in diverse geographic settings. Interviews examined barriers, gaps, successes, and opportunities for integrating gender-informed programming and GBV considerations into outbreak preparedness and response. Authors employed thematic content analysis with a mixed inductive and deductive coding approach to analyze data.

**Results:**

Four major themes emerged. First, participants described the persistent normalization of excluding gender and GBV considerations from outbreak response, driven by competing priorities and inadequate funding. Second, they identified insufficient community engagement and localization, emphasizing that responses often fail to account for existing gender norms, community priorities, and local expertise. Third, participants highlighted fragmented coordination across sectors and organizations, advocating for stronger partnerships with women-led organizations (WLOs), improved collaboration, and more gender-balanced leadership. Finally, they emphasized the need to better institutionalize lessons learned and operationalize existing evidence to strengthen preparedness and support more effective, gender-informed responses during future outbreaks.

**Conclusions:**

To strengthen gender integration in global outbreak response, we recommend embedding GBV risk mitigation throughout response planning, improving cross-sector coordination and gender-balanced leadership, strengthening partnerships with local WLOs, and adopting flexible long-term funding mechanisms. Integrating gender-informed approaches into outbreak preparedness is essential to protecting women and girls, strengthening community resilience, and improving the effectiveness and sustainability of public health responses in LMICs.

**Supplementary Information:**

The online version contains supplementary material available at 10.1186/s44263-026-00305-7.

## Background

Infectious disease outbreaks (“outbreaks”) are increasing in frequency and magnitude globally due to climate change, rapid urbanization, demographic transitions, and global travel patterns [1]. Low- and middle-income countries (LMICs) are particularly vulnerable due to already high communicable and noncommunicable disease burdens, weakened and overburdened health systems, and poor infrastructure and sanitation structures [2]. These pre-existing challenges compound during outbreaks, causing immense physical, social, and economic damage–including increased mortality and morbidity, service disruptions, and gross domestic product (GDP) losses [3, 4].

Outbreaks discriminately impact women and girls through direct and indirect pathways shaped by gender roles, norms, and inequalities. Gender refers to the socially constructed characteristics, norms, behaviors, and roles associated with women, men, girls, and boys, as well as their relationships with one another [5]. Direct gendered impacts of outbreaks include differences in infection risk, disease severity, and care-seeking behaviors, such as women’s heightened occupational exposure due to their representation of approximately 70 percent of the global health and social care workforce [6, 7]. Indirect impacts occur when outbreaks and associated response measures interact with and exacerbate existing gender inequalities, producing adverse consequences across multiple dimensions of women’s and girls’ lives.

Critically, pre-existing gender norms and persisting inequalities shape women and girls’ experiences during outbreaks. Women and girls face increased barriers to health care, financial and economic instability due to gendered social roles, diminished educational and social attainment, and worsened health outcomes (8–10]. Despite experiencing heightened infection risk and worsened outcomes, women often experience limited decision-making power, leaving their needs unmet [11, 12]. These gendered determinants of health and wellbeing underscore the need to integrate gender analysis into public health preparedness and response efforts–both to strengthen intervention efficacy and advance gender and health equity [11].

The COVID-19 pandemic demonstrated how outbreaks and response measures can exacerbate existing gender inequalities. In LMICs, the reallocation of health system resources reduced access to essential sexual and reproductive health services, while lockdowns and school closures disproportionately increased women’s unpaid caregiving responsibilities and economic insecurity and disrupted girls’ education (13–17]. Women also experienced heightened mental health impacts and remained underrepresented in outbreak decision-making bodies, limiting the integration of their needs and perspectives into response planning [18, 19]. Intersecting factors such as race, class, disability, and migration status further compounded these consequences, particularly among women in LMICs and humanitarian settings [20].

Among these gendered impacts, heightened risk of experiencing gender-based violence (GBV) is particularly critical (21–26]. The Inter-Agency Standing Committee defines GBV as “any harmful act perpetrated against a person’s will and that is based on socially ascribed (i.e., gender) roles and power differences between males and females,” including intimate partner violence (IPV), non-partner sexual and physical violence, and child marriage [27]. COVID-19 also highlighted this “shadow pandemic,” as scholars widely claimed an increase in incidence and severity of GBV worldwide and local actors reported significant surges in domestic violence helpline calls and emergency shelter demand [22, 23, 25, 26, 28, 29].

Outbreak response measures—including lockdowns, service closures, and mobility restrictions—can inadvertently increase GBV risk [23, 24, 28, 29]. A recent review identified income loss due to economic shutdown, financial insecurity, and/or job loss; movement restrictions; changes in access to public services; and fear of exposure as four main drivers of violence against women and girls during COVID-19 in LMICs [25]. Response measures can also expand perpetrators’ abusive tactics and force survivors to isolate with their perpetrators, hindering risk mitigation and support access [21, 24].

Community engagement is a foundational component of public health emergency (PHE) preparedness and response [7]. Lessons from COVID-19 demonstrate that building trust and meaningfully involving communities in the design of context-specific interventions are critical to improving uptake and adherence [6, 13]. Effective community engagement is also central to localized, gender-informed responses. Understanding existing gender norms and meaningfully engaging women and girls can help identify barriers, inform mitigation measures, and prevent unintended consequences before interventions are implemented.

Despite growing recognition that infectious disease outbreaks disproportionately affect women and girls, important gaps remain in understanding how gender and GBV are integrated into outbreak preparedness and response in LMICs. This study aimed to examine current approaches to gender integration in outbreak preparedness and response, identify key barriers and opportunities, and generate recommendations to strengthen gender-informed programming and improve the protection of women and girls during future outbreaks.

## Methods

### Recruitment and participants

Ten service providers working in international public health response and/or humanitarian aid in LMICs were interviewed for this study from a total of 28 contacted (Table 1). All participants had 15 + years of experience in outbreak response and programming and worked in intergovernmental organizations. Inclusion criteria specified that participants had in-country experience working on policy and/or response for at least one specific PHE of an infectious disease. Individuals who did not speak English, Spanish, or French were excluded. There were no inclusion criteria related to geography. Prior experience in direct GBV or gender-informed programming was not required for participation. Please see Table 1 for participants’ demographics and characteristics.

**Table 1:** Participant characteristics

Participant ID	Gender	Region of Origin	Organization Type	Field	Outbreak Experience	Regional Experience
**IDI-1**	Male	CWE	UN Agency	Public Health Emergency	Cholera, COVID-19, Ebola	SSA, global
**IDI-2**	Male	NA	UN Agency	Health Emergency Preparedness and Response	Mpox, Diphtheria, Cholera, Marburg, Ebola	SSA
**IDI-5**	Female	NA	UN Agency	Global Emergency Response Team (ERT)	Ebola, Mpox, COVID-19, Cholera	SSA
**IDI-14**	Female	NA	Governmental Organization	Maternal, Newborn & Reproductive Health in Humanitarian Settings	Ebola, COVID-19, Mpox, Marburg, Hurricane response	SSA, SE Asia, LAC
**IDI-23**	Male	NA	NGO / Academia	Emergency Medicine	Bird flu, Ebola, Hepatitis E, COVID-19, Search and Rescue	MENA, SSA
**IDI-29**	Female	NA	Academic / Research Institute	GBV	Diphtheria, COVID-19	SSA, LAC, MENA, S Asia
**IDI-31**	Female	E Africa	UN Agency	Child Protection	COVID-19, Marburg, Ebola	SSA, SE Asia
**PHE-3**	Female	E Africa	UN Agency	Public Health Emergencies	Ebola, COVID-19, Cholera, Marburg	SSA
**PHE-10**	Female	CWE	NGO	Public Health Emergencies	Ebola, Marburg, Cholera, Mpox, COVID-19	Global
**PHE-17**	Male	CWE	Multilateral	Epidemiology and Analytics for Response	Ebola, Mpox, Cholera, Marburg, Plague, COVID-19	SSA, MENA, S Asia, ECA

Abbreviations: NGO = non-governmental organization; UN = United Nations; SSA = Sub-Saharan Africa; SE = South East; S= South; E = East; MENA = Middle East and North Africa; LAC = Latin America and the Caribbean; ECA = Europe and Central Asia; CWE = Central and Western Europe; NA = North America

The sample was identified through a combination of purposive and snowball sampling. Initial interviews were scheduled through UNICEF’s professional networks. The research team then conducted a second round of interviews with participant-referred contacts, asked for at the end of each interview. Each potential respondent was initially contacted via email.

The sample size reflects a purposive, expert-driven qualitative design aimed at generating in-depth insights rather than statistical generalizability. Participants represented a relatively small and specialized population of global health professionals with direct experience in outbreak response and gender-related programming. Follow-up emails were sent to invited participants as part of recruitment until thematic saturation was achieved, with later interviews reinforcing rather than expanding the analytic framework.

Sample size was partially shaped by the snowball sampling process, as participants increasingly recommended overlapping contacts. The determination of thematic saturation was made collaboratively by the research team during ongoing coding and discussion and was consistent with prior qualitative research demonstrating that saturation can be achieved with similar sample sizes in relatively homogeneous expert samples [30]. The final sample of ten key informants was therefore deemed appropriate for the study’s objectives.

### Data collection

Guided by feminist and syndemic theory, the interview guide examined current outbreak response protocols, lessons learned from previous outbreaks, the role of gender in outbreak programming, and GBV prevention and intervention recommendations (Please see Supplementary Material 1 to view the full interview guide) [31, 32]. Participants were prompted to provide examples from past emergency response efforts when possible, discussing experiences with Ebola, COVID-19, Mpox, Zika, and Cholera, among other outbreaks. At the beginning of each interview, the interviewer introduced themselves, described the purpose of the study and data use, and obtained consent for recording.

Participants were encouraged to discuss gender considerations in their responses, including both gender-informed programming and GBV-specific interventions. Gender was a central, a priori focus of the study and was explicitly integrated into the interview guide, with questions directly probing gender roles, norms, and GBV within outbreak response. For example, participants were asked: “Please describe an example of a successful PHE response that you supported. To what extent were gender roles, gender norms, and/or gender-based violence taken into consideration within this response?” and “What are cross-cutting gender considerations that you think all PHE responses should include?”

Interviews lasted between 45 and 75 minutes and took place from October 2024 to January 2025. The research team conducted interviews remotely via Zoom from the US and South Pacific [33]. Despite the availability to conduct interviews in various languages (English, French, and Spanish), all interviews were conducted in English. Four trained qualitative researchers conducted the interviews. Interviews were audio-recorded and transcribed.

This study was conducted and reported in accordance with the Consolidated Criteria for Reporting Qualitative Research (COREQ) checklist [34]. Please see Supplementary Material 2.

### Data analysis

The research team employed thematic content analysis using a mixed inductive and deductive approach with “flexible coding” as per Deterding and Waters [35]. The initial codebook was developed through a review of all transcripts in conjunction with the study objectives and relevant literature. Deductive codes were generated based on the research questions and existing evidence on GBV and gender-informed programming in outbreak settings, while inductive codes were added following a full review of transcripts to capture emergent themes reflected in participants’ responses. Non-verbal cues were captured during the note taking process, which informed memoing and codebook construction.

Using Dedoose qualitative analysis software, three team members coded the transcripts, with two reviewers double coding each transcript [36]. Meetings were conducted throughout the analysis process to resolve any coding questions, problems, or discrepancies. Discrepancies were resolved through consensus and the research team refined the code definitions until all analysts had a shared understanding in code application.

The codebook remained iterative throughout the coding process. While all initial parent codes were retained, additional child codes were introduced to capture nuance and variation within themes as analysis progressed. These additions were discussed and agreed upon through regular team meetings, with discrepancies resolved by consensus. Changes to the codebook were systematically documented through versioning of a shared codebook. In total, the final codebook included 11 parent codes and 40 child codes.

Analytically, gender was operationalized through a set of deductive codes reflecting gender norms, gender roles, GBV integration, and gender-informed programming, all derived from the study objectives and existing literature. These codes were applied across all transcripts to examine how gender was incorporated—or omitted—within participants’ descriptions of outbreak preparedness and response. At the same time, the analysis remained open to inductive insights. Additional sub-themes related to gender (e.g., normalization of gender exclusion, gendered leadership dynamics, and gaps in localization) emerged through iterative coding and were incorporated as child codes. Key informants were not involved in the analysis process and no repeat interviews were needed.

Please find the full codebook within Supplementary Material 3.

### Reflexivity statement

All authors identify as women and have professional expertise in the protection of women and girls in humanitarian settings, with affiliations spanning academia at Washington University in St. Louis and UNICEF. The team is primarily based in the United States (DS, IS, SK, CH, LS), with additional perspectives from authors living in Africa (CP) and the South Pacific (MM). The authors acknowledge that their professional and institutional positions may influence interpretation and analysis and sought to mitigate this through critical reflection and grounding findings in the data and existing literature.

## Results

The ten public health practitioner participants held senior technical and leadership positions in outbreak preparedness and response, and represented United Nations agencies, governmental organizations, non-governmental organizations, academia, and multilateral organizations (Table 1). Each had approximately 15 to more than 20 years of experience and drew on work across multiple infectious disease outbreaks—including Ebola, COVID-19, Mpox, Cholera, Marburg, Diphtheria, Plague, Hepatitis E, and avian influenza—in diverse LMIC settings spanning Sub-Saharan Africa, the Middle East and North Africa, South and Southeast Asia, and Latin America and the Caribbean.

Based on participants’ expertise, four main themes emerged from this analysis relating to GBV, outbreaks, and emergency response. The first theme encompassed a systemic de-prioritization of GBV and gender considerations in outbreak response, where the urgency of contagion and a lack of designated funds often eclipses GBV mitigation efforts. The second theme addressed community engagement and localization challenges during response planning and implementation, identifying current system level barriers and stressing the need for community-informed, tailored programming to effectively impact gender-related issues during outbreaks. The third theme focused on coordination gaps among sectors and actors, with participants advocating for increased local partnerships and diverse leadership to improve GBV response efficacy and sustainability. The fourth theme highlighted the need for institutional learning and the utilization of existing gender-related data to inform outbreak programming and implement evidence-based practice. Please see Table 2 for a summary of key themes and findings.

**Table 2:** Summary of key themes and recommendations related to gender-informed outbreak preparedness and response from qualitative analysis of interviews with public health practitioners in LMICs

Theme	Description	Key Issues Identified	Implications for Practice and Policy
Normalization of gender and GBV exclusion	GBV and gender considerations are consistently treated as secondary to infection control during outbreaks	• Limited funding • Perception of GBV as “non-essential” • Reluctance to prioritize GBV both individually and systematically	• Elevate gender as a core component of outbreak response • Bolster monitoring and measurement • Transition to flexible, long-term funding practices • Increase individual ownership over gender integration and prioritization
Community engagement and localization	Programs often fail to integrate local contexts, needs, and gender norms	• Tokenistic “localization” • Top-down approaches • Insufficient inclusion of affected populations and WLOs	• Shift toward co-created, community-led programming • Strategic partnerships with WLOs
Weak coordination and limited diverse leadership	Fragmented systems and lack of diverse decision-making hinder effective GBV response	• Poor cross-sector collaboration • Gender imbalance in leadership • Exclusion of marginalized voices	• Strengthen coordination and communication mechanisms • Promote gender-balanced, diverse leadership in decision-making roles • Prioritize interdisciplinary collaboration • Strategic partnerships with WLOs
Institutional and shared learning	Existing knowledge and lessons from past outbreaks are not effectively utilized	• Weak knowledge-sharing systems across prior outbreaks and among countries • Underuse of existing data • Repeated mistakes across responses	• Institutionalize learning • Improve data use • Build systems for continuous knowledge transfer and preparedness

Abbreviations: LMICs = low- and middle-income countries; GBV = gender-based violence; WLOs = women-led organizations

### Normalization of gender and GBV exclusion in outbreak response

Throughout interviews, participants referenced what they believed to be a failure of global health responders, local actors, and governments to prioritize and “*systematically address gender issues*” (PHE-10). Participants cited this systemic de-prioritization and a lack of dedicated resources as key barriers to gender integration and GBV response within outbreak programming. Participants described how gender is often seen as peripheral or “*niche*,” rather than as a core component of outbreak response (PHE-10):

*Sometimes there is no focus…on the impact of the public health emergencies on women and girls because of their roles*,* because of their responsibility*,* because of their vulnerabilities and exposure to increased gender discrimination*,* and then of course*,* violence. So*,* it has always been a challenge.* (IDI-5)

Elaborating on this marginality, one participant critiqued a perceived de-emphasis of these issues, stressing that GBV response should be integrated throughout all public health and response areas: “*People don’t think of [gender] as*,* well*,* they don’t think of it at all. It’s not on their radar*,* unless they’re in one of these fields where it plays a huge role anyway*,* like GBV or SRH [Sexual and Reproductive Health]”* (IDI-14). She continued by acknowledging the lack of gender integration and awareness within fields that do not traditionally focus on these issues, mentioning how sectors like “*agriculture*,” “*nutrition*,* or shelter*,” “*don’t necessarily have [these issues] on [their] radar or think that [they] can take that on in any way*” (IDI-14). Citing the systemic nature of this problem, another participant acknowledged challenges leaders face in gender integration when institutions are not built to support these interventions:

*For me*,* this is something I care quite deeply about. And even when I’m in charge*,* I’m setting the direction*,* I’m setting the strategy*,* I’m picking the tools. Even then*,* I find it very difficult to actually say*,* ‘oh*,* I moved the needle on our gender inclusion or our protection approach*,*’ because I find that…what we’re prioritizing at a systems level*,* not in any one institution*,* but at the systems level…the tools that we’re using are so consistently gender insensitive*,* or protection insensitive.* (PHE-10)

Despite holding a leadership position, this participant still felt constrained in integrating gender within outbreak response due to a lack of systems-level prioritization and compatibility. It is difficult for one individual leader to enact change and “*move the needle*” without a broader, systemic push.

Additionally, participants noted that limited resources and funding inhibited GBV and gender-informed responses. They specified that donor’s lack of prioritization of GBV and gender-related issues has caused a major gap in funding to ensure a safer response for women and girls, where actors “*do not have human resource capacity or…financial capacity to be able to implement” *GBV interventions (IDI-5). Participants explained that the implementation of protection measures are also inhibited by donors’ tendency to only fund services directly related to infection:

*Most of the time we do not have allocated resources for*,* you know*,* addressing some of [gender-related issues]. And of course*,* we talk about the consideration*,* but then when the funding comes*,* they go to the sectors that are like hell*,* and sometimes they do not see those as a priority.* (IDI-5)

Another participant expanded on the challenges that accompany this lack of funding, particularly when resources are not secured from the onset: “*If you do not have any form of seed funding at the beginning to allow people to kind of run with these things*,*…you miss the initial opportunity to strike while the iron is hot*” (PHE-3). Without monetary backing, participants felt that gender-related issues could be cast aside.

Despite this systemic lack of prioritization, participants broadly supported the integration of gender-related considerations within outbreak responses, “*mak[ing] sure that this becomes a conversation in all of the meetings that we attend from the onset*” (IDI-5). Another participant recommended efforts to raise actor awareness during outbreaks including “*making sure that health actors understand the potential protection implications that their response may have*” (IDI-31). Participants suggested that wider education on the potential consequences of their interventions and funding—or lack thereof—can mitigate increased vulnerability during outbreaks.

### Community engagement and localization

Challenges to and calls for proper community-engaged and localized programming were common themes during interviews. To effectively understand and intervene in gender-related issues during outbreaks, participants asserted that programs must be rooted in local cultures and their realities, emphasizing a fundamental gap in program design. They advocated for a more nuanced understanding of pre-existing gender norms, values, and beliefs to inform responses and discussed current shortcomings in doing so. One respondent shared:

*We need more visibility and more inclusion. You know*,* we talk about localization and it’s*,* you know*,* so often just lip service*,* and we need to get better at being aware of that so that the gender considerations…have real validity and [are] grounded in those we’re providing services to.* (IDI-14)

Another explained it this way:

*95 percent of risks that are present in the public health emergency*,* the GBV risks*,*…are not new risks. They’re existing risks and they are being reinforced by the emergency*,* right? So*,* an understanding of the local context and its general situation is important to be able to also then adjust the response…I think that’s often forgotten because then we make it look like*,* you know*,* it’s because of the public health emergency*,* but*,* you know*,* the main cause is probably the social norms and the pre-existing levels of violence that are reinforced because of the risk*,* because of the hardship*,* because of the poverty*,* because of the isolation that the public health emergency brings.* (IDI-31)

According to this participant, we must ground gender-informed responses in local contexts to improve protection, intervention, and preparedness. Reflecting on a recent experience from the Mpox outbreak, another participant similarly emphasized the importance of tailoring programming to at-risk groups, yet described a systemic tendency to create one-size-fits-all interventions:

*We know that*,* you know*,* the Clade II [strain of] Mpox is very much related to men [who] have sex with men…And yet*,* we still really*,* really struggle to do adaptive programming*,* to do targeted programming. We still do*,* like*,* lowest common denominator*,* or like*,* the every-man intervention*,* even though we know that these are key drivers.* (PHE-10)

This participant further noted that this limitation is not just the case for stigmatized groups, stating,

*Even when it’s*,* let’s say*,* an easy population*,* like*,* non-sex workers*,* children*,* what have you*,* it’s being spread through children playing in a playground*,* which is very non-controversial*,* it’s still difficult to adapt programming to the specific needs of any given population.* (PHE-10)

Collectively, these accounts highlight a persistent disconnect between recognition of context-specific risk factors and the continued reliance on generalized, insufficiently tailored intervention approaches.

Overall, participants saw community engagement as a key facilitator to creating adaptive, culturally sensitive, gender-informed programming, and participants often stressed the importance of meaningfully engaging communities when creating targeted solutions and designing programs. Participants criticized current approaches that they felt are too frequently “*top-down*,” often simply “*checking the box*” and lacking the depth of collaboration that would foster true co-ownership (PHE-10, IDI-5). One participant described the systemic shift that must occur to improve community engagement in this way:

*There’s a big cultural change that has to happen for experts to say*,* ‘Actually*,* your perspective and your culture and your religion and your whatever matter quite a bit. And my job is to find a way to keep you safe while still meeting your minimum cultural requirements*,*’ and not the other way around. But that’s a big shift. And we’re not there.* (PHE-10)

This participant emphasizes the need for a fundamental shift in priorities and thinking among response actors—from imposing external frameworks toward centering and valuing local knowledge, cultural context, and community-defined needs in designing protective interventions. Building on calls for bolstered community engagement, another participant recommended that public health organizations critically evaluate their criteria for community “*consultations*” (IDI-5). The participant posited that currently,

*they talk to maybe the service providers; they talk to the community leaders. Of course*,* these community leaders*,* majority of them are men…And then we come out with decisions where what services to provide*,* where these services should be located. And then that doesn’t take into consideration what the women needs are.* (IDI-5)

This participant critiques surface-level conceptions and executions of community-based learning. Overall, participants felt that insufficient community engagement can have negative implications on service provision for women.

Participants ultimately encouraged the “*co-creation of epidemic response*” with local actors, including women and other marginalized groups, to ensure that implementations are informed by their key priorities, risks, and preferences, “*because if the direction is set by not locals*,* then it’s not localized*” (PHE-10). Participants further recommended strengthening partnerships with local governments and integrating women-led organizations (WLOs) into response planning to facilitate effective community engagement and programming. One participant discussed how community-based WLOs are often overlooked, despite “*the power that they have…for so many things…making you able to respond to everyone*,* not just to other women*” (IDI-14). Another participant highlighted local WLOs’ value in ensuring preparedness, sustainability of responses, community engagement, and contextual understanding:

*The other piece of it*,* I think*,* that we can also push will be around the partnership…So*,* how do we continue to build capacity of the local organization and particularly for the [WLOs]…because this local organization of [WLOs] are in their communities… So*,* if there is a public health emergency in the future before we even come*,* they are already able to do some maybe preventive measures or some responsive measures.* (IDI-5)

Given that WLOs are embedded within communities and led by those affected, this participant stresses their capacity for impact in bolstering prevention, protection, and response during outbreaks. However, participants did not touch on who would spearhead an improvement in community engagement and tailored program design, begging the question of how this change would happen.

### Gaps in coordination, collaboration, and gender composition in outbreak response

Participants frequently discussed a lack of coordination and collaboration within the global humanitarian architecture, inhibiting efforts to combat GBV and implement gender-informed programming during outbreaks. Participants primarily focused their discussion on GBV efforts, despite being asked about gender considerations more broadly. Because of the interdisciplinary nature of GBV, participants described coordination and collaboration as critical to effective prevention and intervention, where “*protection—GBV considerations—nee[d] to be integrated in all aspects of the health response” *during* “every stage where a person in the context could find themselves*” (IDI-31). Another participant stressed that intervention requires a coordinated approach:

*I think*,* um*,* one of the things is that [GBV] doesn’t fall within one response domain*,* right? … It*,* in itself*,* needs a lot of coordination… there’s the medical side*,* the mental health side*,* and then there’s the sort of community engagement and prevention side…and that needs quite a close collaboration to make it work.* (PHE-17)

The nature of GBV as “*multi-faceted*” requires a coordinated, transdisciplinary response, as well as collaboration and buy-in from multiple sectors.

Despite the importance of coordination, participants described how current approaches to GBV prevention and response during outbreaks are often fragmented by sector and become systemic challenges. One participant warned against a lack of “*cross learning*.” They explained:

*Some of these public health emergencies*,* you know*,* have definitely been also humanitarian emergencies*,* but instead of combining those folks*,* or learning from folks who have been dealing with long standing humanitarian emergencies*,* like we saw in Ebola*,* you have this overlay of a separate system*,* and not learning about the communities*,* the gender issues.* (IDI-14)

While this fragmentation was a common theme, discussed by multiple participants, there were also occasional examples of resource-sharing and coordination, despite existing barriers. When discussing an instance where one organization shared test kits with another in need, one participant shared:

*[Our organization was] able to provide the resources…[this] is not something that happened most of the time in the public health emergency. So*,* how can we make sure that we improve on that so that we are not seeing some of the errors or mistakes?* (IDI-5)

When implemented, participants felt that communication and collaboration among organizations allowed actors to fill critical gaps in GBV response.

To foster collaboration, participants called for increased partnerships with and decision-making power for women and diverse groups in the planning of outbreak response. One participant indicated that an increase of female leaders and personnel would significantly improve the efficacy of response and programming:

*Like a lot of it isn’t kind of a high-tech*,* complicated solution. It’s actually pretty straightforward…a huge piece…is just having more women aid workers. Like more women*,* kind of the face of aid*,* be more female across the board… there’s really a need to like*,* think about kind of how maybe that’s replicating certain harmful gender norms or restrictions.* (IDI-29)

To this participant, the solution is as simple as adding female representation and decision-making power. Echoing the importance of female leadership, another participant highlighted the gender gap endemic in response systems:

*I would include the gender of care providers and responders at different levels because I can tell you in every epidemic I’ve ever been in*,* 90 percent of responders at the community level are women and 90 percent of decision makers at the response level are men.* (PHE-10)

These participants acknowledged the adverse impact of gender imbalances on the implementation of GBV measures and gender-informed outbreak response and the critical importance of the identity and experiences of the leaders making decisions and designing responses. Participants also addressed the need for a diverse workforce, beyond gender, “*because actually the population affected also needs respondents that can relate to several different problems. That’s very*,* very clear in many responses*,* that not everybody can do the same job*” (PHE-17). Without the meaningful inclusion of marginalized voices in decision-making, the needs and priorities of affected communities are unlikely to be adequately recognized or acted upon, perpetuating cycles of sexism and racism within response systems.

### Building institutional learning and drawing on lessons learned

Lastly, participants often discussed the importance of building and operationalizing existing evidence to inform future gender-informed and GBV responses during outbreaks. They emphasized the need for institutional memory and systems that retain and apply lessons across outbreaks and countries. Participants urged actors to learn from past shortcomings because “*we fail and then we fail better each time*” (IDI-1). One participant discussed their organization’s failure to institutionalize past learning of GBV response: “*New staff come in*,* it’s like nothing happened*,* and then it’s like everybody is starting that fresh. So*,* what can we maybe make reference to so that this can be consistent and institutionalized*” (IDI-5). Another participant highlighted the necessity of learning from past outbreaks:

*And then paying even more attention to what we learned going through COVID*,* going through Ebola*,* because people got very creative and innovative*,* and those little lessons learned sometimes don’t make it out of the NGOs where they happened or within their own niche community. And so*,* trying to like glean those nuggets of innovation. (*IDI-14)

They continued to also note the impact of learning from other countries’ responses:

*And of course*,* one size doesn’t fit all*,* but getting the different ideas from how do they do it in Northeast Nigeria and how did they work in[the Democratic Republic of Congo] DRC and etc.*,* and taking all those*,* and trying it*,* and tweaking it*,* and just doing a better job at gathering that learned information to move forward with….but yeah*,* just trying to push forward and keep building that knowledge base and not letting those lessons get lost in the rush*,* in the isolation of responses.* (IDI-14)

Response actors can save precious time, resources, and lives, as well as implement creative problem-solving by learning from past responses, rather than reinventing the wheel every time an outbreak occurs.

Improving institutional learning can also enhance actors’ ability to prepare for increased gender-based risk during future outbreaks. Preparation is especially critical due to rising outbreak occurrence; the lack of time, resources, and capacity during the emergency; and exacerbated urgency and fatality of spread during outbreaks. One participant began explaining the importance of preparedness:

*I also think the preparedness cycle is not done enough. The whole emergency planning*,* emergency response planning*,* building up that resiliency*,* so the hits that communities take aren’t as deep and don’t take as long to recover because we know many parts of the world*,* right*,* are hit with outbreaks over and over and over again.* (IDI-14)

The participant continued, discussing the role of shared learning from past experiences in preparedness:

*So*,* really trying to learn from those places and how to better have plans in place and not just policies at the national level*,* but practices on the ground on. What are you gonna do the next time…there’s an Ebola outbreak*,* like how can we better pre-position things that we don’t get hit so hard next time? That’s another really critical component. And when you’re outside the humanitarian realm*,* I think you have a better opportunity to do that because you’re not operating on six months funding cycles*,* but long-term views of how to deal with these problems.* (IDI-14)

Participants saw collaborative learning and ensuing preparation as a means to combat the constraints of outbreaks. Furthermore, participants posited that past knowledge can not only bolster future responses but also increase awareness of gender-responsive programing. When discussing one organization’s efforts to document lessons learned from an outbreak in Uganda, a participant said,

*the purpose of documenting the lessons learned from the Uganda experience is to actually be able to also support other future public health in emergencies programming and how can we really continue to support country offices… And then that helps to even increase this awareness of what is the impact of women and girls*,* not just from the disease perspective*,* but from the protection and the gender perspective.* (IDI-5)

By raising awareness through lessons learned from previous outbreaks, we can more readily and systematically integrate gender-informed and GBV interventions into all facets of outbreak response.

Similarly, participants discussed shortcomings with current utilization of existing data within systems. One particular participant spent a significant portion of their interview discussing the lack of integration and systemization of data and urged for a drastic improvement of outbreak analysis and definitions of success. Rather than recommend an increase in data collection, this participant stressed the need to bolster actors’ operationalization of existing evidence:

*You’ve invested in understanding the dynamics of why certain populations are more affected or certain behaviors are driving transmission or certain people are more*,* you know*,* more exposed or whatever. But we haven’t invested in really systematizing the thinking about what you do about that.* (PHE-10)

This participant believes that actors adequately understand the social implications of outbreaks and must now shift to systematically employing the evidence to inform solutions. An improvement in the collection, documentation, operationalization, and institutionalization of learning can help prepare for future outbreaks, including the potential exacerbation of GBV prevalence and risk, and reduce significant damage.

## Discussion

This qualitative study aimed to understand global health practitioners’ perspectives on integrating gender considerations within outbreak response in LMICs, with a specific focus on GBV. Four major themes relating to GBV response and gender-informed programming emerged. It is important to note that while interview questions specifically referenced gender, gender norms, and GBV, respondents primarily focused their responses on GBV, possibly reflecting the limited ways in which gender is considered during PHE responses, if at all.

First, participants underscored a persistent de-prioritization of GBV amid the urgency of infection, as well as limited dedicated funds for gender-related issues. Researchers across multiple outbreaks, including Ebola and Zika, have shown a pattern in which gender considerations are deprioritized in emergency response [11, 15]. During the Zika epidemic, limited attention to gender reinforced harmful norms and placed women at greater risk, as they were blamed for contagion [37]. Similarly, a recent systematic review reported a dearth of research on GBV occurrence during outbreaks and an absence of rigorous research that explored gendered violence during any outbreak other than COVID-19 [25].

Similarly, public health research and standardized PHE protocols often prioritize WASH, Infection Prevention and Control (IPC), and clinical management, treating GBV and other social harms as secondary considerations and ignoring their value as core analytical variable [38, 39]. This myopic approach creates a critical operational blind spot: actors implement interventions under the assumption of gender-neutrality, failing to account for potential harms to community safety and a disproportionate impact on women and girls [38].

Insufficient and inconsistent funding and capacity during outbreaks has also been a longstanding and well-documented barrier to GBV-specific programming, with less than one percent of humanitarian funding allocated to the GBV sector (40–42]. Studies have shown a trend of persistent underfunding to gender equity and GBV prevention and response initiatives globally, despite proven returns of funding [43, 44]. Participants stressed that funding cycles that prioritize short-term results can hinder gender-informed and GBV response. Rather, a switch to long-term, flexible funding structures can facilitate resilience during the constrained conditions of outbreaks, investment in sustainable GBV-related solutions as necessary, and the prioritization of increasing and emerging GBV-related needs [13, 17, 18, 27]. These issues are increasingly important to contend with as funding and resources continue to dwindle [27, 45, 46].

It is important to note that the integration of gender-informed programming into outbreak response does not inherently require additional funds—it is rather at the heart of effective program design from the onset, involving an intentional prioritization of gender inclusion and ownership among actors to implement these changes. While participants emphasized funding gaps and structural challenges, many protection measures require design changes rather than additional resources. As such, GBV- and gender-related gaps may relate less to capacity and more to priorities embedded in program design. To create effective change, actors and leaders within organizations and governments must take ownership of these initiatives, consistently prioritizing gender throughout program design, implementation, and evaluation.

Expanding systematic GBV monitoring during various types of outbreaks could further bolster actor and donor knowledge, as well as willingness to fund and implement gender-informed programming. Development of existing measurement tools could support the promotion of GBV-related knowledge among relevant actors. A review, systematically mapping existing humanitarian tools for GBV, found that while numerous resources exist, most lack clear guidance on monitoring outputs, integration in existing monitoring and evaluation frameworks, and outcomes of GBV risk mitigation activities [6]. The improvement of standardized and accessible measurement approaches can provide critical data points that strengthen awareness, accountability, and effectiveness of GBV risk mitigation during PHEs, as well as reduce the burden of data collection for smaller organizations [6, 24].

Secondly, participants critiqued current conceptualizations of localization and community engagement as top-down and deficient, emphasizing the critical importance of embedding pre-existing cultural norms, values, and contexts in GBV response and gender-informed programming. These findings are mirrored in the literature [42, 47, 48]. It is well established that community-led responses—guided by affected communities—can introduce trust, establish lines of communication, support cost-effective service delivery, and reach marginalized groups when public and private institutions cannot [19, 47, 49, 50]. They empower host communities to participate in addressing their own needs and health disparities, set appropriate agendas, and take ownership [51].

However, despite its proven efficacy, it is well documented that few global health interventions properly engage communities, much less implement gender-informed, community-led programming [35, 40, 41, 52]. Similarly, within child protection in developing contexts, top-down approaches have been characterized by low formal service utilization and nonformal-formal service misalignment, while slower, community-driven, and bottom-up approaches have enabled social change, formal service utilization, and higher levels of community ownership [20]. 

WLOs and other community-based organizations can strengthen the efficacy and sustainability of emergency responses by contributing context-specific expertise, local embeddedness, and flexibility (52–54]. Bolstering these approaches requires greater community ownership and inclusivity, integration with existing structures and processes, and engagement with local authorities [55].

Third, given the pervasive reach and impact of gender-related issues, participants urged for improved coordination between sectors of international organizations and amongst international, national, and local actors to improve breadth, scope, and efficacy of programming. Weak coordination of GBV and gender-informed responses contributes to fragmented programming, documented to undermine the efficiency and resilience of health interventions and hinder sustainable improvements in global health [40, 56].

GBV especially–as a wide-reaching, transdisciplinary issue–requires coordinated efforts to ensure effective protection and response during outbreaks [41]. Across socioecological levels, community- and government-led efforts are synergistic and indispensable parts of preparedness and response [42, 47]. Findings and literature support a push towards stronger cross-sector and -actor coordination mechanisms and transdisciplinary problem-solving to ensure that GBV considerations are systematically integrated into outbreak preparedness and response [49, 57].

Outbreak response is often medicalized, prioritizing disease containment over broader social and gender-related consequences. Although the importance of integrating gender and GBV considerations is recognized, these priorities may be sidelined during acute response efforts. A medicalized approach, which fails to recognize the socioeconomic impacts of both outbreaks and response measures, can exacerbate existing inequalities [58, 59]. Our findings therefore reinforce the need for multisectoral leadership that extends beyond traditional expertise in epidemiology, WASH/IPC, clinical management, and health products to incorporate gender, protection, and other disciplines critical to community wellbeing [60]. While prioritizing disease containment during an emergency is essential, a singular focus on controlling transmission risks overlooks the potentially severe and fatal indirect consequences of outbreaks and response measures.

Furthermore, gender imbalances in outbreak decision-making are well documented, with women holding only five percent of leadership positions in global health organizations in LMICs [40, 53, 61]. Such underrepresentation can undermine effective programming and increase risks for women and girls, reinforcing calls for more diverse and gender-balanced governance to support gender-inclusive outbreak responses [40, 53, 62]. Strengthening the roles of women and diverse groups can ensure cultural relevance, sustainability, effective decision-making, and improved outcomes within global health programming [44].

Lastly, certain participants emphasized the utility of building institutional and shared learning from past outbreaks and countries to bolster GBV preparedness, program sustainability, and awareness. Scholars have widely called for structural improvements to facilitate knowledge sharing and improved preparedness, including a long-term commitment to enhanced data collection efforts, data repositories, and stakeholder convenings [63]. Research on the COVID-19 pandemic and this study’s findings posit the importance of collaborative learning and accountability, urging actors to proactively identify and learn from past successes and failures and initiate the institutionalization of best practices for pandemic response [50].

Across themes, participants identified interconnected shortcomings and opportunities to strengthen the protection of women and girls during outbreaks, with each finding reinforcing and compounding the others. The de-prioritization of GBV underpins gaps in coordination, limits meaningful community engagement, and constrains the use of data and institutional learning, while the lack of institutional learning and diverse leadership interferes with an emphasis on gender integration during future outbreaks. Together, the themes illustrate a reinforcing system of barriers, while also providing a roadmap for integrated, gender-informed programming, grounded in real-world experience.

Following analysis, authors vigorously debated onus of responsibility for systemic transformation. Although participants acknowledged the importance of the integration of gender-informed responses and community engagement, no participant took accountability over or suggested a plan forward for the implementation of these changes. Participants attributed GBV de-prioritization to ‘the system’ without critically interrogating their role within the exclusion of gender- and GBV-related issues and their complicity in perpetuating harmful systems. Although it can be difficult to enact change on the individual level, this discrepancy may suggest that the issue extends beyond coordination mechanisms or funding structures to encompass the values, priorities, and perhaps unconscious biases of decision-makers themselves. Addressing GBV and integrating gender within outbreak response may require not only structural changes but also confronting how patriarchal norms shape professional cultures, program design choices, and what responders perceive as ‘essential’ services.

Building on these findings, we propose interconnected recommendations to strengthen gender-informed outbreak preparedness and response (Table 3). Together, these actions provide practical, systems-level opportunities to address the structural barriers identified by participants and support more equitable and effective outbreak responses. Implementation of these recommendations can improve gender-specific protection, preparedness, and response during outbreaks in LMICs.

**Table 3:** Actionable recommendations to improve the protection of women and girls during outbreaks in LMICs

Recommendation	Rationale from Findings	Key Actions	Key Actors
Promote gender-balanced and diverse leadership	Greater representation of women and marginalized groups can improve prioritization of gender and GBV throughout preparedness and response	• Structural changes in hiring and promotion • Uplift traditionally marginalized voices in key decisions and program planning	Governments, UN agencies, NGOs
Strengthen partnerships with WL and community-based organizations	Local organizations provide contextual expertise, strengthen community trust, and improve sustainability of interventions	• Collaborate with community-based organizations and integrate WLOs into response plans • Incorporate leadership incentives on WLO partnerships and women’s empowerment • Bolster research on the impact of WLOs’ leadership on GBV mitigation during outbreaks and effective community-based planning and programming	Governments, UN agencies, NGOs, Researchers
Improve GBV monitoring, measurement, and accountability	Standardized indicators and monitoring can strengthen evidence, accountability, and investment in gender-informed programming	• Integrate standardized GBV indicators into outbreak monitoring and evaluation frameworks • Routinely collect and report GBV risk mitigation outcomes throughout preparedness and response • Invest in harmonized, user-friendly measurement tools that strengthen accountability and reduce reporting burden	Researchers, Governments, Humanitarian Organizations
Adopt flexible, long-term funding mechanisms	Sustainable funding enables preparedness, institutional learning, and continued GBV prevention and response beyond acute emergencies	• Adopt flexible, multi-year funding mechanisms that support preparedness, sustained GBV programming, and adaptation to evolving outbreak needs • Increase direct funding to WL and community-based organizations to strengthen sustainable and context-specific outbreak responses	Donors, Governments
Prioritize localized, community-engaged planning	Meaningful engagement with affected communities improves cultural relevance, trust, and intervention effectiveness	• Integrate meaningful community participation from the outset with clear guidelines and evidence-based best practices • Establish mechanisms for community members to meaningfully shape program priorities based on local needs and contexts • Conduct rigorous evaluations of community-engaged approaches to strengthen the evidence base and support their systematic adoption	Governments, UN agencies, NGOs, Local Organizations, Researchers

Abbreviations: LMICs = low- and middle-income countries; WL = women-led; WLO = women-led organizations

To our knowledge, this study is among the few pieces of literature to offer empirically grounded, cross-sector expert perspectives on the status of gender integration within outbreak response in LMICs and key recommendations to improve the protection of women and girls within these settings. Findings identify not only structural barriers—such as funding, coordination, and data gaps—but also highlight deeper issues in decision-making norms, accountability, and the persistent disconnect between knowledge and implementation. Importantly, the study contributes actionable insight by emphasizing the need to shift power toward localized, women-led actors and to embed gender considerations into core response design—not as an add-on, but as fundamental to effective outbreak preparedness and response.

While the themes that emerged from our analysis were well-developed and supported by the data, the study’s findings should be interpreted in light of certain limitations. While the snowball approach broadened the pool of potential participants and our sample size is validated in the literature given the study aims, our sample was relatively small affecting generalizability.

Further, although purposive sampling through UNICEF’s networks allowed our team to effectively and efficiently reach key informants in the field, this method may have introduced sampling bias. Participants’ experiences and responses may reflect specific institutional norms and policy alignment, potentially influencing findings. However, the breadth of participants’ expertise, jobs, organizations, and geographic and infectious disease focus provided a variety of perspectives. It is also important to note that several respondents worked for organizations other than UNICEF at the time of the outbreaks (Table 1).

Responses may also be influenced by social desirability bias, reducing participants’ critical reflection on shortcomings or personal accountability. Finally, the absence of community members or GBV survivors in our sample limits the study’s ability to capture lived experiences. Despite these limitations, the study design was well-suited to its aims, enabling the capture of experienced, cross-sector insights that are critical for understanding systemic gaps and informing actionable improvements in outbreak response.

## Conclusions

This study provides empirically grounded, cross-sector practitioner insights into the persistent barriers to integrating gender and GBV considerations into outbreak preparedness and response in LMICs. Findings demonstrate that gender and GBV remain deprioritized, insufficiently localized, poorly coordinated, and inadequately informed by existing evidence and lessons learned, reflecting a persistent gap between knowledge and implementation. Recommendations emphasize embedding gender and GBV considerations throughout outbreak preparedness and response through diverse leadership, meaningful community and WLO partnerships, strengthened monitoring and accountability, flexible long-term funding, greater actor ownership, and localized, gender-equitable programming. Addressing this gap is critical to advancing more equitable, effective, and contextually responsive outbreak preparedness and response that better protects women and girls.

## Supplementary Information

Below is the link to the electronic supplementary material.

Supplementary Material 1

Supplementary Material 2

Supplementary Material 3

## Data Availability

Raw data is not provided to protect participant confidentiality. Data may be available upon reasonable request to the authors. For inquiries, please contact lindsaystark@wustl.edu.
