# Supplementary material for: Outbreak preparedness for women and girls in low- and middle-income countries: a qualitative study
Source: BMC Glob Public Health. 2026 Jul 28;4:70. doi: 10.1186/s44263-026-00305-7 (PMC13417842; doi:10.1186/s44263-026-00305-7)
Supplement: Supplementary file 2 — Supplementary Material 2 [file 44263_2026_305_MOESM2_ESM.docx]

Supplementary Material 1: Interview Guide

# Annex 1: CHRNI Survey

*Introduction:*

Thank you for your willingness to participate. As we may have mentioned previously, we are interested in public health emergencies (PHE), particularly infectious disease outbreaks. We want to understand how gender considerations, such as gender norms and roles, may impact the successes of PHE responses.

To help us better understand how gender considerations are and are not incorporated into PHE responses, we are holding several interviews with different experts to understand their unique and shared experiences. We appreciate your interest in participating in this interview given your experience and insights. The interview will focus on your own experiences and perceptions of PHEs and their corresponding response measures, to support the first step in identifying research gaps and priorities on this topic. We anticipate this interview will take around 30 to 45 minutes of your time.

After the interviews, we will transcribe the audio recordings, identify common themes and knowledge gaps, and develop these into a list of potential research questions. Then, we will ask for your participation in a brief online survey to help prioritize the identified areas of research. Your input is very valuable to us. We want to ensure that setting a learning agenda is a collaborative process that is led by contributions of experts like you. Before we get started, do I have your permission to record our conversation?

*Research Question:*

How do gender roles, gender norms, and GBV impact the success of PHE responses?

*Interview Questions:*

1. Please describe your experience working on public health emergencies.
   1. *[Probe]:* Ask about geographical location, job title, organization, responsibilities, as well as extent that responsibilities directly involve GBV AND/OR PHE programming, policy, or response.
2. Can you tell me about some of the public health emergencies (specifically, infectious disease outbreaks) that you have worked on?
   1. [*Probe*]: Ask for details about the public health emergencies and responses (geographic focus, population, timeline, specifics about the measures or guidance put in place, etc.)
   2. [*Probe*]: Ask about the response development process, both what was experienced and the perceived ideal approach.
3. Looking back on these public health emergencies, to what extent is gender (norms, roles, violence) considered within PHE and corresponding responses?
   1. [*Prompt*]: We are interested in understanding how gender impacted the success of control measures or how control measures considered gender. Some things that we have heard about include [INSERT EXAMPLE FROM EARLIER INTERVIEWS]. Have you had similar experiences?
4. Generally speaking, what are the current strengths of PHE responses in regard to considering gender roles, gender norms, or gender-based violence?
   1. *[Prompt]*: How has this changed across time and between PHEs?
5. Please describe an example of a successful public health emergency response that you supported.
   1. *[Probe]:* Ask about specifics regarding the context of the response and the response itself
   2. *[Prompt]:* To what extent were gender roles, gender norms, and/or gender-based violence taken into consideration within this response?
   3. *[Prompt]:* Please describe if/how any of these gender considerations could be replicated or considered beyond this specific PHE response?
6. What are common mistakes that PHE responses make when considering gender roles, gender norms, or gender-based violence?
   1. *[Prompt]*: How could these mistakes be avoided or mitigated?
   2. *[Prompt]*: Can you give an example of a time when there was a failure to take gender into account as part of a PHE response?
7. What are cross-cutting gender considerations that you think all PHE responses should include?
   1. *[Probe]*: Follow up on gender roles, gender norms, and/or GBV
   2. *[Prompt]:* In what ways do you think [X GENDER CONSIDERATION] would have on the success of the PHE response?
8. Are there other things that would help improve the success of PHE responses?
   1. [*Probe*]: Ask about tangential resources, programming, knowledge, training, or other gaps that could be filled.
9. We are interested in speaking with other experts like you and getting their input and ideas as well. Are there any other experts who you think we should connect with?
   1. *[Prompt]: If yes, can you please provide their names and contact information?*
10. Is there any other information that you want to share with me?

*[Thank the participant for the time shared and let the participant know that an email from our team will be shared soon with further instructions on the prioritization exercise]*
