# Supplementary material for: Outbreak preparedness for women and girls in low- and middle-income countries: a qualitative study
Source: BMC Glob Public Health. 2026 Jul 28;4:70. doi: 10.1186/s44263-026-00305-7 (PMC13417842; doi:10.1186/s44263-026-00305-7)
Supplement: Supplementary file 3 — Supplementary Material 3 [file 44263_2026_305_MOESM3_ESM.pdf]

Supplementary Material 3: Codebook

Acronyms
GBV – gender-based violence
IDO – infectious disease outbreak
PHE – public health emergency
IPV – intimate partner violence

Codebook

Code	Child Code	Description	Example
Association of GBV with IDOs	Change in prevalence/incidence	Discussion of a change in prevalence or incidence of GBV due to an IDO.	
	Change in frequency	Discussion of a change in frequency of GBV due to an IDO.	
	Change in severity	Discussion of a change in severity of GBV due to an IDO.	
	Change in location	Discussion of a change in location of GBV. For example, girls being more likely to be alone when outside, so more vulnerable to nIPV.	
	Change in typology	Discussion of a change of typology of GBV. For example, increase use of manipulation relating to infection spread and increase in digital violence.	
	Other change	Any other change in GBV due to IDO.	
Factors influencing GBV change during IDO	Lockdown	Discussion of a change in GBV due to lockdown during an IDO.	
	Job loss	Discussion of a change in GBV due to job loss during an IDO.	
	Cultural norms	Discussion of a change in GBV due to cultural norms during an IDO.	"Yeah, I think for COVID, the thing it depends on exactly the on the, on the background of say there's, you know, cultural norms of the country itself. Because umm we we saw the disruption in in education. Yeah, you know, in some countries, you know, a lot of women, actually, girls, I mean, a lot of girls actually drop off completely, you know, went into early marriage." (IDI 2) (co-coded with school closures)
	School closures	Discussion of a change in GBV due to school closures during an IDO.	"So you increase the risk, uh, by taking such measures. When you close schools is another example, because it is documented that there is, uh,

			associated risk of gender based violence when school are being closed with the risk of uh not going back to school or then having forced or having my marriage increase in marriage associated and etc, etc." (IDI 1)
	Closure of non-essential services	Discussion of a change in GBV due to the closure of non-essential services during an IDO.	"you know, people are worried that they may get the disease they go to hospitals. And of course, we have umm let's say, reproductive health services that are also not accessible. So so this, you know, can create some, you know, unintended, yeah, I would say gender issues because women don't have access to reproductive health services." (IDI 2)
Past Responses to IDOs	Successes	Discussion of any success of past IDO responses, relating both to disease spread and GBV response.	"the concept of SBV, of gender-based violence, is a is a very sensitive one to bring to govern. And of course, depending on the on the government, some governments are more sensitive to it umm than than others. But I think UNICEF has been doing a good job in kind of men's training those aspects in the response." (IDI 2)
	Failures/ Shortcomings	Discussions of any failures or shortcomings of past IDO responses, relating both to disease spread and GBV response.	
	Lessons Learned	Discussion of lessons learned from past IDOs and how they were documented.	
Recommendations	Funding	A recommendation for increased funding for GBV efforts in a future IDO.	"I think there is something that needs to be further unpacked about the wider gender based violence that a public health emergency response might create, might amplify or amplify the risk or actually create direct, uh, maybe gender based violence, uh, mechanism or, um, or, uh, the, the insufficient, uh, investment in having, you know, gen- services that can identify and respond to gender based violence in the response in our programs." (IDI 1)
	Protocol	A recommendation for changed/updated protocols for GBV efforts in a future IDO.	
	Evidence-based Toolkit	A recommendation for toolkits for GBV efforts in a future IDO.	"That's a bit, sometimes a bit missing because we work based on our experience, but this is usually biased because it's our own experience. So something that is more systematic and maybe more evidence based on how to look at this and respond. Then the, the, the other thing is how this work that you can do can inform maybe some, uh, some tools to see how we can apply a gender lens systematically on the different component of a public health emergency response." (IDI 1)
	Research	A recommendation for increased research regarding GBV efforts/programming/ prevalence in a future IDO.	

	Training	A recommendation for increased training on GBV efforts in a future IDO.	
	Community Engagement	A recommendation for increased community engagement for GBV efforts in a future IDO.	
	Essential Services	A discussion on changing the essential service designation or protocol in future IDOs to account for changes in GBV.	"I think, uh, uh, uh, starting to have a better understanding or, or collective awareness that you need to have a continuity of essential health, social services, and humanitarian assistance, and a stronger community engagement and, uh, being part of an outbreak response or infectious disease public health emergency response, and that was that has happened. I would say already during the response in, in, in, in DRC, and then it's insufficiently happened during the COVID 19 global response, but it was part of what was discussed and there was an effort toward that." (IDI 1) (co-coded with community engagement)
	Integration of GBV response in other services	A recommendation for the integration of GBV responses in other services during a future IDO.	"This is uh, a requirement to be made that each public health and social measures that is taken for our outbreak control needs to have, uh, a gender, an analysis of what are the gender implication and the risk of gender-based violence associated, which we did not have this. as part of the, of the response. So that's one. Um, that's an example." (IDI 1)
	Data collection & documentation	A recommendation for increased data collection and documentation on GBV occurrence and pathways in a future IDO.	"So, I think the primary, the primary, you know, advocacy, uhh yeah I would say, pitch for UNICEF during any outbreak, is to make sure that we have genders, you know, sex disaggregated data." (IDI 2)
	Personnel/leadership	Recommendations on personnel and leadership staff to address GBV in a future IDO.	
	Areas of improvement	General areas for improvement in a future response, necessitating a solution (without mention of a specific recommendation).	
	Government policies	A recommendation for changes in government policies for GBV efforts in a future IDO.	
Interdisciplinary nature of GBV response		Discussion of the interdisciplinary nature of GBV response.	"I think one thing that has been made very clear in especially the 2018 DRC Ebola outbreak is that because it happened in a very specific context, which was a humanitarian, an existing humanitarian crisis, is that it could not be disconnected from other, uh, programs that were already in place for humanitarian assistance to the affected population. And that the two response were in influencing each other in, especially the fact that the public health and social measures that could be put in place for the

			infectious disease control could have a serious impact in disrupting humanitarian assistance to the population." (IDI 1)<br><br>"So, these are stuff that UNICEF is is known to be good at, and of course, there's one uhh specific pillar of response that's probably of interest to you, that's kind of cross sectoral that every single UNICEF response has to integrate uhh aspect of uhhh gender, you know, -based, violence related. So, this is how it is. And yep, uhh once where the plan is there, we we just support the government to to response." (IDI 2) (co-code GBV response changes)
	Intentionally interdisciplinary	Discussion of an interdisciplinary GBV response that was intentionally implemented.	
	Inadvertently interdisciplinary	Discussion of an interdisciplinary GBV response that was inadvertently implemented.	
Call to Action		Discussion of a personal or organizational motivation to start or continue working on GBV efforts within IDO response.	"We, we did have system in place with training of the staff and with all the checklist of what needs to be done. And despite that this happened. Uh, which means that there is something more that was, that needs to be done or changing the systems. And I genuinely think that at least in UNICEF at the management level, everybody had some awareness of SEA (Sexual Exploitation and Abuse) and wanted to prevent, but despite all this, this happened, which means that the systems, uh, have not been able to deliver on this." (IDI 1) (co-code with areas of improvement)
Gender norms		Any discussion of gender norms, relating to either response or spread.	
	Gender inequality	Discussion of gender inequality, relating to either response or spread.	"For each position we had, I don't know the statistic, but the proportion of male applicants versus women's applicant females applicants were- and I'm making my own, but it was in this order. It was nine male for [1] female. Um, it can be explained by the fact that it was, we were requesting for immediate availability. We were requesting to go in very hard, uh, settings and for relatively long, uh, availability. And all these are influenced by pre-existing gender dynamic in the society." (IDI 1)
	Role of gender in ID spread	Discussion of the role of gender, contributing to ID spread.	"You took some good examples that is gender sensitive, simply because, you know, the the the the women, let's say that they they take care of the sick, so they are more, they are kind of highly exposed. Some, some, some differences in a in a number of cases." (IDI 2)
Role of gender in programming		Discussion of the role of gender within IDO responses.	"For each position we had, I don't know the statistic, but the proportion of male applicants versus women's applicant females applicants were- and I'm making my own, but it was in this order. It was nine male for [1]

			female. Um, it can be explained by the fact that it was, we were requesting for immediate availability. We were requesting to go in very hard, uh, settings and for relatively long, uh, availability. And all these are influenced by pre-existing gender dynamic in the society." (IDI 1)
	Intentional inclusion of gender within programming	Discussion of an intentional inclusion of gender within IDO responses and programming.	
	Inadvertent inclusion of gender within programming	Discussion of an inadvertent gender lens within IDO responses and programming or an inadvertent effect of gender on programs.	
	Gender overlooked within programming	Discussion of a lack of awareness or acknowledgement of gender within IDO responses and programming.	
Sustainability		Discussion of sustainability of programming or lack thereof, relating to current or the need for future efforts.	
Current response protocols		Any discussion of current response protocols, relating either to ID or GBV prevention/mitigation.	"So, umm what will, what the country will do, will also, will also do our own risk assessment and based on some simple criteria, you know, like umm, you know, 'what is the what is the risk of expansion of the outbreak? What is the risk to to to children? What are the country capacities to response?' You know, 'what are the risks associated with the country's mutual essential services?'" (IDI 2)
	GBV response changes	Discussion of new implementations of protocols, relating to GBV, based on increased knowledge. In other words, lessons learned and implemented.	"We've noticed in some of the outbreaks that is extremely important that to have gender sensitive, let's say isolation facilities. So, some diseases you have to isolate umm cases, you have gender sensitivity related to quarantine of of the cases, you know, we have seen that in some of the outbreaks. There are some diseases where you have uhh your exposure." (IDI 2) (co-code with role of gender in programming)
Good quote		Any instance of a good quote that should be captured in the findings.	
